# News and Coming Events

**Published:** 1907-08-24

**Authors:** 


### NEWS AND COMING EVENTS.

The Cottage Hospital at Fyvie, which has been completely overhauled, and to which a new wing has been added, was opened in the first week of August by Lady Leith of Fyvie.

It is proposed to enlarge and reconstruct the Weybridge Cottage Hospital. The scheme provides for fourteen beds altogether, and allows at a slight increase of cost for the development of four more. The total estimate is under £4,000.

At the last meeting of the Board of Managers of the Swansea Hospital it was announced that the working men's contributions to the hospital funds during the financial year amounted to £3,799 out of a total of £10,000, while house-to-house collections had yielded £459 odd.

The Royal Westminster Ophthalmic Hospital, King William Street, W.C., has received a grant of £1,000 from the Trustees of the Zunz Bequest for the purpose of naming a ward, in perpetuity, the "Annie Zunz Ward."

The new wing of the Royal Isle of Wight County Hospital was opened by H.R.H. Princess Henry of Battenberg early in the present month. The new buildings have been erected from designs of Mr. Thomas Cutler, the contractor being Mr. Henry Cawte, of Shirley Works, Southampton.

A new cottage hospital has been opened at Ramsey in the Isle of Man. The building consists of two wards, each of four beds and two cots, with two single bed wards, a nurses' room, store room and sanitary conveniences. Attached to these is a neat little surgery and operating room.

The Visiting Committee of the Prescot Board of Guardians has recommended the erection of a new hospital for mental, epileptic, and infectious cases at Whiston Workhouse. After some discussion the Guardians decided to vote a sum of £9,000 to be expended in building and equipping an extension to the general hospital as recommended.

The carnival recently held in aid of the Ilford Hospital has proved highly successful, it being estimated that the building fund of the hospital, which already amounts to over £2,000, will be augmented by £700 as the result of the collections. A special feature of the carnival was the excellent work done by the children, who, dressed as nurses, assiduously levied contributions from everyone.

A physician of Omaha, Nebraska, four years ago submitted his diploma to the Nebraska State Medical Board at Lincoln. The document was posted back to him by express, but never reached him, and as Harvard College refuses to issue duplicate diplomas, the doctor is suing the express company for damages to the amount of £5,000, claiming that the loss of his certificate will prevent him practising in any other State.

At a special general meeting of the managers of the Northern Infirmary at Inverness a letter was read from Dr. Mackay, of Inverness, offering a sum of £1,200 for the purpose of "equipping and maintaining the treatment of phthisis on modern lines." After discussion and report by the medical committee, it was decided to accept the offer, and to provide for the establishment of a consumption ward, either by extending the present west wing of the infirmary or by building a separate ward.

Princess Louise, Duchess of Argyle, will open the new wing of the Leicester Infirmary on November 5 next.

At the annual meeting of the Clayton Hospital and Wakefield General Infirmary some interesting facts were disclosed with reference to the consumption of alcohol in the hospital. In 1874, when the infirmary had only 14 beds, the alcohol bill totalled £39 and the milk bill £45. Ten years later, with 34 beds, £53 was spent on alcohol and £165 on milk. Since then the patients have had far less alcohol, though their number has been progressively increasing, so that the milk bill for last year approached close to £300.

Scare legislation is common enough in America, but even drastic temperance reformers will find the latest development of temperance legislation, as exemplified by the "Prohibition of the Sale of Alcoholic Beverages Act," which has just become law in Georgia, a trifle too aggressive. Under this Bill no intoxicating beverage of any kind can be legally sold in the State of Georgia after January 1, 1908. Druggists may only dispense prescriptions which contain alcohol upon a written order signed by a qualified practitioner. Such prescriptions must be made up immediately on the day they are written or upon the following day, and a special book, open to public inspection and somewhat on the lines of a poison book, must be kept by every druggist. It will be interesting to see how far this Act will modify intemperance in the State of Georgia.

The Bitter Cry of the Sundayless.—The National Hygienic League, an organisation which has done pioneer service in the direction of securing a legalised six days' working week and equally good work in the suppression of juvenile smoking, has just issued a booklet, from the pen of Mr. T. Bowick, detailing the hardships of those who are unable to afford a Sunday's rest. Professor Sims Woodhead, in a few introductory remarks, urges the introduction of a Weekly Rest-day Bill, and the data given in pamphlet are sufficiently important to prove that such a measure will be warmly welcomed by a large section of the community. The importance of a weekly rest-day is gradually better appreciated. To some, it is true, the religious principle appears to be the predominant factor in influencing their approval of such rest, but it is safe to say that by far the larger majority look upon the subject purely from a practical point of view. Medical men would be the first to support such a measure as the National Hygienic League proposes. To them Sunday is often the hardest day in the week, simply because a certain class of patient prefers to be visited on that day. There are practitioners—and we are glad to say that the number of them is daily growing larger—who set their faces sternly against unnecessary Sunday calls, charging a slightly higher rate for Sunday visits; but the average practitioner feels in duty bound to make no difference between Sunday and week-day calls. We venture to think that if the profession generally made it clear that they, as well as tram drivers, City men, and railway employés, are in need of a breathing space after six days' strenuous labour, their patients would admit the reasonableness of this wish, and do their utmost to ensure its realisation. Urgent calls, of course, will always claim the doctor, but the percentages of urgent Sunday calls is relatively small, and the majority of visits that the "Sunday doctor" has to pay are needless. The general practitioner might do worse than bring Mr. Bowick's booklet to the notice of his patients.

